# German translation and cross-cultural adaptation of the Vestibular Schwannoma Quality of Life Index (VSQOL)

**DOI:** 10.1186/s41687-024-00778-w

**Published:** 2024-08-13

**Authors:** Mareike Rutenkröger, Svenja Wandke, Jens Gempt, Lasse Dührsen, Maximilian Scheer, Christian Strauss, Hannah Führes

**Affiliations:** 1 Department of Medical Psychology, University Medical Center Hamburg-Eppendorf, Hamburg, Germany; 2 II. Department of Medicine, University Medical Center Hamburg-Eppendorf, Hamburg, Germany; 3 Department of Neurosurgery, University Medical Center Hamburg-Eppendorf, Hamburg, Germany; 4 Department of Neurosurgery, University Hospital Halle, Halle (Saale), Germany

**Keywords:** Vestibular schwannoma, Acoustic neuroma, Quality of life, Patient-centeredness, VSQOL

## Abstract

**Background:**

Vestibular schwannomas (VSs) are benign tumors of the vestibulocochlear nerve that often cause significant neurological and functional impairment. Patient-reported outcomes, including quality of life (QoL), are essential for understanding the overall impact of VS and its treatment. This study aimed to translate and culturally adapt the Vestibular Schwannoma Quality of Life (VSQOL) Index into German to expand its relevance to German-speaking populations.

**Methods:**

We used a qualitative approach including translation and cognitive interviews with 10 patients who underwent VS surgery. The translation process followed the TRAPD protocol to ensure linguistic and conceptual accuracy. Cognitive interviews assessed the comprehensibility and relevance of the translated questionnaire.

**Results:**

The translation showed remarkable consistency between translators, with minor discrepancies resolved by consensus. Cognitive interviews provided valuable insights that led to refinements in item wording. Participants emphasized the importance of an additional item on physician referrals, reflecting differences in health care systems between the United States and Germany.

**Conclusions:**

The German VSQOL provides a comprehensive tool for assessing QoL in patients with VS that integrates patient-centered dimensions. A Validation study is underway to establish its reliability and validity.

## Introduction

Vestibular schwannomas (VSs), benign tumors of the vestibulocochlear nerve, often result in significant neurological and functional impairments that affect overall well-being due to disease progression or treatment interventions [1]. While traditional clinical outcomes focus primarily on facial nerve function, hearing preservation, and tumor control, patients often highlight additional factors such as dizziness, pain, fatigue, cognitive issues, and satisfaction or regret with treatment decisions as critical aspects affecting their social and emotional well-being [2–4].

In addition, the integrative model of patient-centeredness emphasizes several dimensions that are central to ensuring an optimal healthcare experience. These dimensions include recognizing each patient as a unique individual with specific needs and preferences, empowering patients to actively participate in their health care decisions and management, and promoting meaningful involvement in their care processes [5]. This framework highlights the importance of recognizing the mismatch between healthcare provider priorities and patient values in quality of life (QoL) research.

The disease-specific Penn Acoustic Neuroma Quality-of-Life (PANQOL) scale was developed in 2010 [6], which has greater sensitivity to relevant disease-specific domains compared to general QOL measures, such as the SF-36 [7] or the Glasgow Benefit Inventory [8]. However, most cross-sectional and prospective studies using the PANQOL scale have failed to demonstrate clinically significant differences between management strategies. Presumably, inherent limitations in study methodology (e.g., inadequate power), inadequate sensitivity of the instrument, or a true lack of a clinically meaningful difference between treatments for the domains under study could account for the lack of detected differences in these studies [9–12].

Drawing on extensive VS QOL research, clinical evidence, and stakeholder feedback from organizations and patients, Carlson et al. [13] identified notable shortcomings in previous instruments, including the omission of crucial domains such as pain and cognition, and the neglect of treatment-related satisfaction or regret. To address these shortcomings, they present the Vestibular Schwannoma Quality of Life Index (VSQOL), a novel disease-specific QOL measure tailored to sporadic VS.

The aim of this study was to translate and culturally adapt the VSQOL into German, thereby extending its applicability and relevance to German-speaking populations.

## Methods

### Study design

We conducted a qualitative study aimed at testing a German VSQOL. Therefore, we developed a German version of the VSQOL through translation and adaptation of the English version. We sought and received the consent of the authors of the original questionnaire to translate the VSQOL in January 2023. The study was approved by the local Ethics Committee of the Center for Psychosocial Medicine of University Medical Center Hamburg Eppendorf (No. LPEK-0665).

#### Translation

We translated the existing English versions of the VSQOL [13] into German using the team translation protocol known as TRAPD (Translation, Review, Adjudication, Pretesting, and Documentation) [14]. This method, which is becoming increasingly recognized in translation research, ensures accuracy and reliability [15]. Initially, two experienced members of our study team (HF, a psychologist experienced with qualitative research; SW, a trained psycho-oncologist), fluent in both German and English, independently undertook the translation process. Next, a third bilingual team member (MR, a psychologist experienced with VS symptoms and treatment) carefully reviewed the translations provided by HF and SW, selecting one or suggesting a third version if necessary. In addition, we sought the expertise of two chief neurosurgeons during the translation process (JG, CS). Finally, HF, SW, and MR engaged in extensive discussions, deliberating on all translations and suggestions until a consensus was reached on the final translation of the VSQOL, ready for further testing to ensure comprehensibility.

#### Adaptation

We conducted cognitive interviews testing the prefinal version of the German VSQOL with patients diagnosed with VS who had undergone surgery. Cognitive interviewing, a method used in cross-cultural adaptation studies, aims to pretest translations to ensure that the content is understood as intended by the author [16–18]. Because of the convenience sampling approach used, theoretical saturation was neither intended nor achievable [19, 20]. However, we observed saturation in the feedback and suggestions provided by our participants. Participants were recruited through the neurosurgical outpatient clinics of the University Medical Center Hamburg, the University Medical Center Halle (Saale), and the German self-help organization *“Vereinigung Akustikusneurinom e.V.”*. Participants were personally invited to participate in the interviews by a member of the study team (HF). An interview guide was developed based on the recommendations of Willis et al. [16, 20], and focused on assessing comprehension of the translated titles, introductions, scale names, and endpoint labels, as well as the 40 items. Verbal probing techniques such as comprehension probes (e.g., “What does the term “quality of life” mean to you?”) and paraphrasing (e.g., “Can you rephrase this sentence in your own words?”) were used. Participants were also asked for suggestions for further improvements of the questionnaire. Demographic and clinical data were collected and descriptive statistics were calculated using SPSS [21]. Participants were offered compensation of 15 Euros. Interviews were conducted via phone by HF, audio recorded, and transcribed verbatim. HF and MT then discussed the results and selected the final version of the questionnaire that was best understood and found most useful by the majority of participants.

## Results

### Translation

The translators (HF, SW, MR) were remarkably consistent in their translations, with only minimal discrepancies noted. The deviations that were observed were mainly minor differences in sentence structure or individual word choices that did not change the overall meaning. We quickly reached a unanimous consensus on the translation of the VSQOL during our first round of discussion. To facilitate the cognitive interviews, we combined different translated versions of individual items in our interview guideline. Specifically, we evaluated two alternative versions for items 10, 14, 21, 22, 26, 30, 35, and 36.

### Adaptation

We conducted cognitive interviews with 10 patients diagnosed with VS. The mean interview duration was 43.77 min. Demographic and clinical data are summarized in Table 1. The majority of participants were over 50 years of age (80%), with an even gender distribution (50% female, 50% male) and predominantly native German speakers (90%). Educational backgrounds varied, with 40% having a low, 40% a medium, and 20% a high level of education. Most of the patients (80%) had a diagnosis less than 5 years ago and 80% had surgery within the last 5 years. The most prevalent preoperative symptoms included tinnitus, dizziness, and hearing loss, while headaches and facial paresis additionally emerged as a common postoperative symptoms.

Participants reported that the introduction and instructions, as well as the measurement scales, were easy to understand. In the adaptation process of the items, certain English terms, like “head fullness” (item 12), presented a challenge due to the absence of direct equivalents in German, consequently resulting in their exclusion from the item. We further engaged patients in discussions about the wording of individual items, focusing on whether they were perceived as factual questions or reflections of subjective experiences, as in the case of “I feel that my healthcare team listens to my concerns (…)” versus “My healthcare team listens to my concerns (…)” (item 36).

The introduction and most of the items were well understood by patients. Recognizing that the term “management”, although meaning the same in German, is not commonly used in medical encounters with patients, we replaced it with “treatment” and added clarification in parentheses to indicate that this includes surgery and/or radiation as well as the “wait and scan” approach. Most patients indicated a preference for wording that captured subjective experiences when describing their physical condition. Conversely, they preferred wording that posed factual questions for items related to treatment decision making.

We chose the item versions preferred by most participants and slightly modified the wording and grammatical structure of sentences according to participants’ suggestions. Therefore, we adapted items 10, 14, 15, 21, 22, 26, 30, 33, 35, and 36 (see Table 2). Participants suggested that item 40 (“I had to stop working altogether and go on permanent disability.”) from the original VSQOL questionnaire should lead the “Impact on Employment” domain to improve coherence, as subsequent items relate to current employment status. They suggested that patients who had stopped working due to illness might want to skip other questions within this subscale. However, we decided not to change the order of the items, as the original authors expressed concern that such changes, such as individuals skipping subsequent questions, would pose potential analytic challenges.

Furthermore, participants emphasized the importance of including an additional question at the end of the questionnaire asking whether their physician had made referrals to other health care providers, such as physical or speech therapists. They also suggested including an open-ended section for additional comments. However, accommodating these requests would significantly alter the original questionnaire.

The scoring instructions were not included in the cognitive interviews, as they are only relevant to those who are tasked with analyzing the questionnaire results.

## Discussion

In this qualitative study we introduced the first German version of the VSQOL. Consensus on the translation of the existing English VSQOL questionnaire was quickly reached during our initial discussions. Through cognitive interviews, we refined the German version to ensure its clarity and relevance for patients with VS who have undergone surgery.

The insights gained from the cognitive interviews with patients provided valuable feedback on the comprehensibility and relevance of the translated questionnaire. Participant input highlighted areas that required adaptation to improve clarity and alignment with patient experience. In particular, participants’ preference for subjective wording regarding their physical state underscored the importance of accurately capturing individual perspectives. Furthermore, the adaptation process facilitated refinements to item wording of the questionnaire. Participant feedback guided these adaptations to ensure coherence and relevance to the target population.

The patients wish to include an additional item asking about physician referrals to other healthcare professionals, such as physical therapists or speech therapists, sheds light on the differences in healthcare systems between the United States and Germany. In the United States, health care is often fragmented, with patients having access to different specialists and allied health professionals [22]. Referrals to physical or speech therapists are common, especially for conditions such as VS, where rehabilitation and speech therapy are critical. In contrast, the German healthcare system operates under a social health insurance model, with primary care physicians often acting as gatekeepers to specialist care. Referrals to allied health professionals may occur, but are typically centralized through primary care physicians, in contrast to the decentralized U.S. system.

Participants in our study unanimously endorsed the importance and relevance of each item to their health-related QOL and unique circumstances. With this collective endorsement, the German VSQOL now serves as a comprehensive tool for assessing QOL in VS patients, integrating patient-centered dimensions. We are currently actively involved in the validation process of the German VSQOL with German and Swiss subsamples to verify its reliability and validity. The translated questionnaire can be obtained from the original authors [1].

**Table 1:** Demographic and clinical data of participating patients of cognitive interviews

	Participants of cognitive interviews (*N* = 10) *n* (%)
Age	
< 30 years	1 (10)
31–40 years	0 (0)
41–50 years	1 (10)
> 50 years	8 (80)
Gender	
Female	5 (50)
Male	5 (50)
Native language	
German	9 (90)
Other	1 (10)
Education^a^	
Low	4 (40)
Medium	4 (40)
High	2 (20)
Time since diagnosis	
< 5 years	6 (60)
5–10 years	1 (10)
> 10 years	3 (30)
Time since surgery	
< 1 year	4 (40)
2–5 years	4 (40)
< 10 years	1 (10)
< 20 years	1 (10)
Preoperative symptoms^b^	
Tinnitus	7 (70)
Dizziness	6 (60)
Hearing loss	7 (70)
Headaches	3 (30)
Facial paresis	0 (0)
Other^c^	3 (30)
Postoperative symptoms^b^	
Tinnitus	8 (80)
Dizziness	8 (80)
Hearing loss	9 (90)
Headaches	7 (70)
Facial paresis	6 (60)
Other^c^	4 (40)
Tumor size (in Koos^d^)	
1	1 (10)
2	3 (30)
3	1 (10)
4	5 (50)

^a^Low < 10 school years, medium = 10–13 school years, high > 13 school years

^b^More than one answer possible

^c^Pre- and postoperative symptoms included cognitive impairments, such as concentration difficulties and word-finding problems

^d^Koos grading system: 1 = tumor is located exclusively in the internal meatus acusticus, 2 = tumor bulges up to the cerebellopontine angle without involving the brain stem, 3 = tumor fills the entire cerebellopontine angle, 4 = tumor displaces the brain stem and the nearby cranial nerves

**Table 2:** Original and adapted items for the German version of the VSQOL

Original Items	Adapted Items
10. Because of my dizziness or imbalance, I do not feel confident while driving	10. I don’t feel safe driving because of my dizziness or balance problems
14. Pain associated with my condition interferes with my daily activities	14. The pain caused by my illness restricts me in my daily activities
15. My tinnitus makes it difficult for me to concentrate	15. My tinnitus affects my ability to concentrate
21. I feel that my overall health is poor	21. I have the impression that my general state of health is poor
22. My condition interferes with my daily activities	22. My illness restricts me in my daily activities
26. My condition has negatively impacted my outlook on life	26. My illness has a negative impact on my view of life
30. I have difficulty finding the right words when speaking or writing	30. I have difficulty finding the right words when I speak or write
33. I feel as if my brain has slowed down	33. I have the impression that my brain has become slower
35. I feel I received enough unbiased information to make a good decision about how to manage my vestibular schwannoma	35. I received enough unbiased information to make a good decision regarding the treatment of my vestibular schwannoma
36. I feel my healthcare team listened to my concerns and preferences when providing a recommendation for how to manage my vestibular schwannoma	36. The treatment recommendation sufficiently addressed my concerns and preferences for the treatment of my vestibular schwannoma

## Data Availability

The datasets used and/or analysed during the current study are available from the corresponding author on reasonable request.

## Abbreviations

VS: Vestibular schwannoma
QoL: Quality of life
VSQOL: Vestibular Schwannoma Quality of Life Index
PANQOL: Penn Acoustic Neuroma Quality-of-Life
